# Injury Patterns In Low Intensity Conflict

**Published:** 2009-12

**Authors:** V Saraswat

**Keywords:** Militancy, Low intensity conflicts, Road traffic accidents (RTA), Trauma, Injuries

## Abstract

**Summary:**

Injury patterns and their outcome has been the subject of interest in all kinds of military conflicts. This retrospective study was conducted in a tertiary care hospital (Level I trauma centre) to find out the trends in injuries in low intensity conflict, adequacy of pre hospital treatment, mortality patterns and adequacy of treatment after reaching tertiary care hospital. 418 patients were treated over a period of two years. All were male and 76% younger than 30 years of age. 61% patients reported directly from the site of incident and 39% were transferred from other trauma centre. Two-third of patients (73.9%) reported with at least one limb injury and 44.9% with extremity injury alone. Multiple injuries were most common injury (29%). Head and neck injuries were seen in 20% patients and Thoracic and abdominal injuries were seen in 2.6% and 3.4% patients only.

Most common mode of injury was Gunshot wound (41.4%), followed by splinter injuries (39.2%) and Road traffic accident(RTA) (19.4%). Overall mortality was 3.8% and inpatient mortality of 1.4%. Head and neck injuries were leading cause of death followed by thoracic injuries.

## Introduction

Trauma from conflicts has been the part of human evolution since beginning of civilization. First road traffic death, on 17th August 1896 at Crystal Palace, London, UK, added a new dimension to the trauma. As the speed of automobile increased, so did the frequency and severity of injuries. Military trauma also kept pace with the development of more powerful and deadlier weapons and protective gears to deal with changing weapons systems. This lead to the change in patterns of injuries and at present is the result of the interplay of weapon design, speed of movement and protective gear used.

With the conventional wars becoming rarer, low intensity conflicts has become the norm of the day; thereby heavy casualties can be inflicted in terms of loss of human life and collateral damage (loss of property and business) without politically declaring a war, at much reduced cost. Trauma in low intensity conflicts involving civil population could bring injuries not usually seen in civil setup.^1^ Injury patterns in defence forces could be different; young, trained men being involved in aggressive military operations.

There is paucity of literature in reporting of injury patterns among militancy /war affected personals and many more focus on affected civilian population. There is definitely a need to know the injury patterns among security personals. This retrospective study was undertaken to find out the trends in injuries in low intensity conflict, adequacy of pre hospital treatment, mortality patterns and adequacy of treatment after reaching tertiary care hospital.

## Methods

All patients admitted to a tertiary care hospital (Level I trauma centre) located in militancy affected area, over a two year period, were included in this retrospective study. Road traffic accidents (RTA), directly related to militancy, were also included in the study.

Level IV trauma care was provided by buddy or trained nursing staff at the site of occurrence, whereas Level III care was provided by field hospitals at a shorter distance. Field triage was carried out and patients transferred to Level II or Level I Trauma centre. Air transport facilities were also available as and when needed. Trauma not related to militancy was not included in the study. Patients admitted for follow up or repeat surgeries were also not included in the study.

All patients were received in a trauma ward, by the trauma team, consisting of surgeon, anaesthesiologist, emergency doctor, ward nurse and other paramedical staff. The team was present in the hospital at all times. On arrival of patient, primary survey was carried out on all patients as per ATLS protocols. Venous access with a wide bore cannula, 16/18G, was secured, blood for all routine, biochemical investigations and grouping and cross matching was drawn and initial resuscitation started. Simultaneously, requisite radiological investigations were carried out. All patients, not requiring surgery, were given definitive treatment and after due stabilization, were shifted to the respective wards. The patients requiring immediate or emergent surgery were sent to operation theatre and results of the investigations were conveyed in the operating room. Rests of the patients were shifted to operation theatre depending on their clinical requirement. Concepts of triage and golden hour were followed. Secondary and tertiary survey was carried out in the respective wards.

Patients were grouped as per the part of the body involved into head and neck, thorax, abdomen, extremities and multiple injuries. Multiple injuries included injury to one extremity along with at least one more part of the body. Patients were also analyzed for type of the injury (fire arm used), requirement of the surgery on arrival to the hospital and analyses of mortality.

Data are presented as absolute numbers, percent- ages or mean.

## Results

In all, 418 patients were treated over a period of two years. All patients were male and 76% younger than 30 years of age. 254 (60.8%) patients reported directly from the site of incident and 164 (39.2%) were transferred from other hospitals (Level II trauma centre) after initial management.

**Fig 1:**
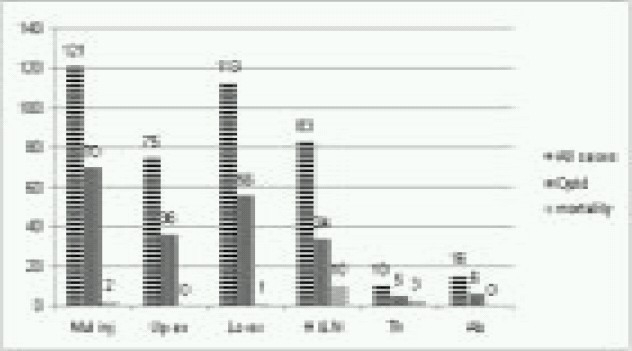
Distribution of injuries

Multiple injuries were most common injury involving 121(28.9%) patients followed by single lower limb injuries in 113(27%) patients. Head and neck injuries were seen in 83(19.9%) patients and 75(17.9%) patients sustained single upper limb injuries. Thoracic and abdominal injuries were seen in ten (2.6%) and sixteen (3.4%) patients only. (Fig-1)

**Fig 2:**
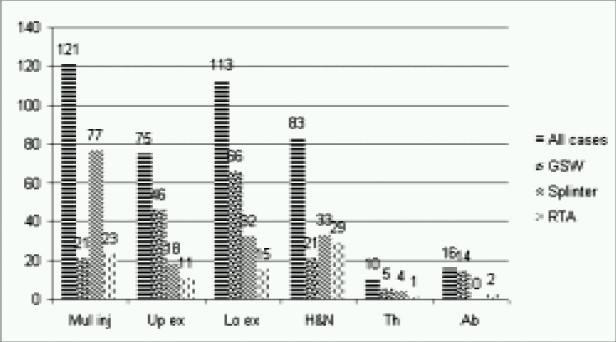
Distribution of mode of injuries

**Mode of injuries-**Gunshot wound was the most common mode of injury, (n=173, 41.4%), followed by splinter injuries from improvised explosive device (IED), grenades and other fire arms (n=164, 39.2%). A significant number of patients (n=81, 19.4%)) were injured in RTA. (Fig-2)

**Injuries to extremities** – Isolated upper and lower limb injury was seen in 75(17.9%) and 113(27%) patients respectively with overall injuries accounting to 188(44.9%). 92(48–9%) patients were operated immediately on arrival. (Fig-1) 112(59.6%) patients suffered as a result of GSW, 50(26.6%) from splinter injuries and 26(13.8%) from RTA. (Fig-2)

**Head and neck injuries** – Head and neck injuries were the second largest group of injuries after extremities (n=96, 23%). Isolated head and neck injuries were seen in 83 (19.9%) patients and another 13(3.1%) along with other limb injuries. 34(41%) patients were operated immediately on arrival. (Fig-1) From isolated injuries, 21 (25.4%) were sustained from gun shots, 33 (39.7%) from splinters and 29 (34.9%) from RTA. Thirteen patients (10 head and neck and 3 eye) also suffered as part of multiple injuries. (Fig-2)

**Fig 3:**
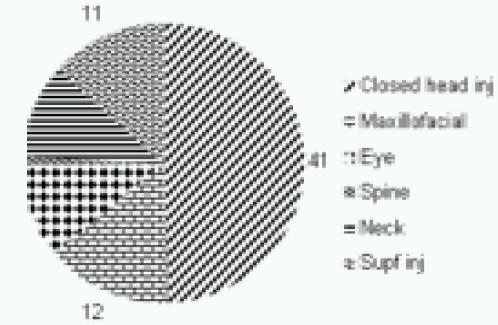
Distribution of head & neck injuries (83)

Closed head injuries accounted for nearly half of them (n-41, 49.4%). Isolated maxillofacial injuries were seen in 12(14.5%) patients and 5 more in association with other injuries. Isolated eye injuries were seen in 9(10.8%) patients and three in association with other injuries. Spine injury was seen in one patient only. Isolated neck injuries were seen in nine patients (10.8%), out of whom three had sustained injuries to major vessel, trachea or larynx; rest did not have any life threatening injury. Eleven patients (13.3%) suffered only superficial injuries. (Fig-3)

**Thoracic injuries** – Isolated thoracic injuries were seen in 10 patients (2.4%) and an equal number had associated limb injuries. Five (50%) needed immediate surgery on arrival and two had already been attended at previous trauma centre, rest three were received ‘Brought-in-dead’.(Fig-1) From isolated injuries five were sustained from gunshots, four from splinters and one from RTA. (Fig-2)

**Abdominal injuries** – Isolated abdominal injuries were seen in 16(3.8%) patients and another six in association with other injuries. Six (37.5%) were operated immediately on arrival and rest had already been dealt with at earlier centre. (Fig-1) Fourteen of the isolated injuries were reported from gunshot and rest two from RTA. (Fig-2)

**Fig 4:**
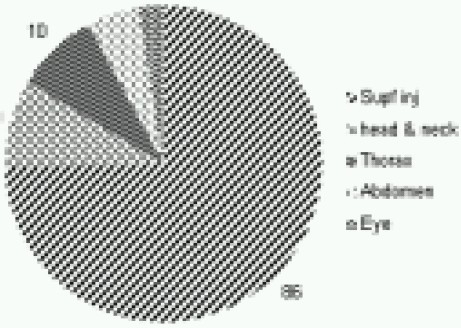
Distribution of Multiple injuries (121)

**Multiple injuries** - Multiple injuries were most common injuries (n=121, 28.9%) (Fig-1). According to the mechanism of injuries majority had sustained splinter injuries (n=77, 63.6%); Gunshot wound and RTAs accounting for 18.2% (n=22) each. (Fig-2) 70 (57.9%) of them needed immediate surgical attention and rest were managed conservatively. (Fig-1) Parts, other than the limb involved, were head and neck and chest (ten each), abdomen (06), eye (03) and multiple limbs (09). Eighty six patients (71%) had suffered superficial injuries involving various parts of the body but did not involve any particular organ system or region. (Fig-4)

**Mortality** - Sixteen patients died from injuries related to militancy trauma with an overall mortality of3.8% during the two year period (Fig-1). Majority of deaths (n-10, 62.5%)) were from head and neck injuries followed by thoracic injuries (n-3, 18.8%) and multiple injuries with head injury (n-2, 13%). Only one death was observed from GSW thigh.

Out of sixteen ten were received Brought-in-dead at the hospital (seven head injuries and three chest injuries), hence true inpatient mortality being only 1.4%. Three patients (all head injuries) expired in first 24 hours and rest three (two multiple injuries with head injury and one GSW thigh) survived more than 24 hours.

No mortality was seen in isolated upper extremity injury, abdominal injuries or multiple injuries.

## Discussion

The military tactics determine the patterns of injuries and its management, more so with the involvement of civilian population, due to element of surprise, unpredictability and lack of protection. Injuries from suicide bombers, in civil population, require tailored approaches and close collaboration and cooperation.^1^ Most of the studies have reported both civilian and military causalities, in contrast to present study being purely on defence personnel.

The speed of transfer of trauma victim to trauma care facility is key in ultimate outcome of the patient. Two different methods of transfer of trauma victims have been suggested; one, ‘Scoop and scoot’ other ‘Stay and stabilize’. Earlier one is ideal for urban settings, with short distances and good transportation facilities whereas later, more helpful in difficult terrains and long distances. Another concept, ‘In-transit stabilization’, constitutes resuscitation during transfer to trauma centre. The choice primarily depends on facilities available for transfer, distance to trauma centre and severity of trauma. The paucity of level II trauma care, in Kosovo, was not felt to be a significant problem, primarily because of short distances and easy availability of both ground and air transport.^2^ Hence speed of transfer to trauma centre is more important than level of trauma care facility.

Prehospital management of patients with severe trauma should focus on control of hemorrhage, stabilization of vital signs and early transport to a trauma centre. Immobilization of cervical spine and maintenance of oxygen delivery are the primary and most important intervention.^3^ Advanced life support (ALS) procedures can be performed by paramedics on major trauma patients without prolonging on-scene time, but they do not seem to improve survival.^4^ There is no convincing evidence that prehospital ALS in the urban setting provides any benefit to injured patients in terms of either morbidity or mortality.^5^ The OPALS Major Trauma Study showed that implementation of full ALS did not decrease mortality or morbidity for major trauma patients and mortality was greater among patients with Glasgow Coma Scale scores less than nine.^6^ This has also been validated in Triage guidelines issued recently.^7^ In present study, ALS procedures were not employed in any patient transferred from site of incidence or Level I trauma centre. Injuries found on patients, ‘Brought -in-dead’ or died within 24 hours, were largely not sustainable with life, severe head injury being single most common cause of mortality followed by thoracic injuries. Hence, enrouteresuscitation may be a better proposition logically.

Triage of patients on the principals of “the greatest good for the greatest number” based on model of urgent, immediate, delayed, minimal, or expectant care is time tested in mass casualties. Expectant care is reserved for victims whosesurvival is unlikely even in the presence of adequate resources.^8^ Criteria for ‘Field Triage Decision Scheme’ has been revised in 2006 guidelines.^7^ Normally triage area is recommended to be established in emergency department.^9^ However, in present setup, the trauma centre was separate from emergency dept and except for one representative (for documentation purpose), was not involved in management and left to deal with regular emergencies.

Early surgery is the mainstay of treatment in trauma and ultimate outcome depends on this single factor. Ideally, ‘Golden Hour’ concept should be followed; however it was not feasible, in such circumstances, at all times. Instead earliest possible surgery was attempted on arrival to a trauma care centre at all levels, which varied from less than an hour to six hours. Nearly half the patients (49.5%) were taken up for surgery immediately on arrival to this tertiary care hospital, maximum being 57.8% from multiple injuries and least (37.5%) from abdominal injuries.(Fig-1) Considering 39% patients were received from Level II trauma care centre majority needed some kind of surgical intervention.

The patterns of injuries may vary considerably depending upon the type of the fire arms used, terrain and type of military operations. Small arms and IEDs accounted for nearly 81% of all injuries and rest injured in RTA. Peleg has reported 95% injuries as a result of small arms and explosive devices in hospitalized terrorist victims, when not taking RTAs in to account.^10^ Zouris has reported 75% injuries to small arms and explosives in US marines in Iraqi war.^11^ Findings in present study are consistent with these two studies. In contrast, Appenzeller has reported two-thirds injuries attributable to blunt trauma and only one-third to combat-type injuries; seventy-four percent of blunt injuries due to MVAs (motor vehicle accidents), accounting for 47% of overall trauma.^2^ This high incidence of blunt trauma and MVAs was attributed to no licensing authority, poorly maintained small roads, lack of traffic control and virtually nonexistent use of seatbelts.

Extremity wounds and fractures traditionally comprise the majority of traumatic injuries in armed conflicts.^12^ Two-third of our patients (73.9%) reported with at least one limb injury and 44.9% with extremity injury alone. Multiple injuries were the most common injuries in the present series (28.9%). Majority were due to firearms (82%) and rest from RTA. Appenzeller, in Kosovo war, also reported extremity injuries to be the most common injuries occurring in 54% of all patients, with 33% of patients presenting solely with extremity injuries.^2^ Zouris, in Iraqi war, reported 70% of all injuries to upper and lower extremities, a percentage con- sistent for battlefield injuries since World War II.^11^

Head and neck injuries were the second largest group of injuries (23%) with nearly half of them being closed head injuries. Loss of consciousness with coup and countercoup injuries formerly were considered secondary or tertiary injuries, but with the increased use of body armour in the military, damage to the central nervous system after an explosion has been increasingly attributed to the direct effects of the blast.^13^,^14^ The higher incidence has been reported from Kosovo war; forty-four percent of all patients having head and neck injuries, whereas only 29% had isolated head and neck involvement.^2^

Thoracic and abdominal injuries were seen in 4.8% and 5% patients and isolated injuries only in 2.4% and 3.8% patients. All injuries were sustained from firearms except one thoracic and two abdominal injuries from RTA. The higher incidence has been reported from Kosovo war; Twenty-one percent having chest injuries with 6% having injuries isolated to the chest and overall, 13% having abdominal wounds, with 4% being isolated abdominal wounds.^2^ This difference could primarily be due to use of body armour by the troops in our series and more RTAs and civilian causalities in Kosovo war. Body armour has been shown to protect military personnel from most ballistic projectiles to the torso, thus increasing survival. However, it does not protect against the barotraumas of primary blast injury.^15^

Mortality may be taken as one indicator of adequacy of prehospital resuscitation. Trunkey (1983) identified three separate peaks of trauma deaths immediate, early and late.^16^ However in mass causalities from disaster and blasts, high immediate and low early and late mortality has been reported. The present series show an overall mortality of 3.8% and an inpatient mortality of 1.4%. (Fig-1) Peleg has reported inpatient mortality of 6%.^10^

Preparedness can reduce morbidity and mortality considerably and can improve response and outcomes.^17^ This has been proved in various disasters. California, with a high preparedness index, had a low case fatality rate, approximately one per 100 injuries, from the 1989 Loma Prieta and 1994 Northridge earthquakes. Kobe, Japan, with mixed levels of preparedness had a case fatality rate of 31 per 100 injuries subsequent to the 1995 earthquake. At the other extreme, the 1988 Armenia earthquake, where there was a low preparedness index, produced a case fatality rate of 137 per 100 injuries.^17^ This may be true in low intensity conflict also and high level of preparedness may improve outcome in trauma victims.

Injuries from low intensity conflict are marked by surprise and varied in nature. They require early transport to trauma centre and comprehensive surgical management. Pre-hospital stabilisation of patient before transfer is not mandatory; however in-transit resuscitation may be helpful. Control of haemorrhage, cervical spine stabilisation and oxygen supplementation are most important interventions in pre-hospital care. Mortality depends more on severity of injury and speed of transfer to hospital than aggressive pre-hospital management. Trends from present series suggest high extremity and head injuries. Although injuries from fire arms and explosive cannot be mitigated, RTA can be further reduced with better traffic discipline. There is a requirement of high degree of awareness, good transportation facilities, health care preparedness and quick reaction on occurrence of incident.
